# Analysis of Wave Patterns Under the Region of Macro-Fiber Composite Transducer to Improve the Analytical Modelling for Directivity Calculation in Isotropic Medium

**DOI:** 10.3390/s20082280

**Published:** 2020-04-17

**Authors:** Kumar Anubhav Tiwari, Renaldas Raisutis, Liudas Mazeika

**Affiliations:** 1 Ultrasound Research Institute, Kaunas University of Technology, K. Baršausko St. 59, LT-51423 Kaunas, Lithuania; renaldas.raisutis@ktu.lt (R.R.); liudas.mazeika@ktu.lt (L.M.); 2 Department of Electrical Power Systems, Faculty of Electrical and Electronics Engineering, Kaunas University of Technology, Studentu g. 50, LT-51368 Kaunas, Lithuania; 3 Department of Multimedia Engineering, Kaunas University of Technology, Studentu g. 50, LT-51368 Kaunas, Lithuania

**Keywords:** wave patterns, analytical model, directivity pattern, guided wave (GW), non-destructive testing (NDT), macro-fiber composite (MFC), transducer

## Abstract

Analytical modelling is an efficient approach to estimate the directivity of a transducer generating guided waves in the research field of ultrasonic non-destructive testing of the large and complex structures due to its short processing time as compared to the numerical modelling and experimental techniques. The wave patterns or the amplitude variations along the region of ultrasonic transducer itself depend on its behavior, excitation frequency, and the type of propagating wave mode. Depending on the wave-pattern of a propagating wave mode, the appropriate value of the amplitude correction factor must be multiplied to the amplitudes of the excitation signal for the accurate evaluation of directivity pattern of the ultrasonic transducers generating guided waves in analytical modelling. The objective of this work is to analyse the wave patterns under the region of macro-fiber composite (MFC) transducer to improve the accuracy of a previously developed analytical model for the prediction of directivity patterns. Firstly, the amplitude correction factor based on the wave patterns under the region of P1-type MFC (MFC-2814) transducer at two different frequencies (80 kHz, 3 periods and 220 kHz, 3 period) glued on 2 mm Al alloy plate has been estimated analytically in the case of an asymmetric (A0) guided Lamb wave. The validation of analytically estimated amplitude correction factor is performed by a proposed experimental method that allows analyzing the behaviour of MFC transducer under its region by gluing MFC on bottom surface and scanning the receiver on the top surface of the sample. Later on, the estimated amplitude correction factor is included in the previously developed 2D analytical model for the improvement in the directivity patterns of the A0 mode. The modified analytical model shows a significant improvement in the directivity pattern of the A0 wave mode in comparison to the results obtained by the previous model without considering the proper wave patterns. The results reveal that errors between the directivity estimated by the present modified 2D analytical model and experimental investigation are reduced by more than 58% in comparison to the previously developed analytical model.

## 1. Introduction

One of the key issues in the structural health monitoring (SHM) of various composite structures and components is to maintain the safety, reliability, and operational performance [1,2,3]. For the last few decades, ultrasonic guided waves (GWs) have been used for this purpose to detect and locate the defects in the structures. Among all the available non-destructive testing (NDT) techniques, ultrasonic guided wave (GW) testing has been the most promising due to its high sensitivity to the defects and wide coverage region [4,5]. Moreover, GW testing is fast, can cover up the defective regions to reasonable distances, and has the ability to detect defects underground, water, or a layer of insulation [6,7,8]. In comparison to guided wave testing, bulk wave testing is tedious and time-consuming, requires high-level training, uses the point-by-point scanning method, and needs a visible area and accessibility of the defective region [9,10]. Due to the high sensitivity of GWs to the variation in modulus of elasticity (E) of the material under testing and minimal amplitude damping of propagating wave modes, only a few measurements are required for the inspection of large infrastructures to detect internal and surface defects [1,10,11]. Researchers have successfully utilized GWs for inspecting defects/damages in metallic structures [12], concrete structures [13,14], pipes [15,16,17], and composite structures [18,19,20,21,22,23,24].

The Lamb wave is a specific type of guided wave that propagates in a plate-type structures and can be further categorized into the symmetric Lamb waves (S0, S1…) and asymmetric Lamb waves (A0, A1…) depending on the value of frequency-thickness product (*f*·*t*), *f* is the excitation frequency of ultrasonic transducer and *t* is the thickness of propagating medium or structure under inspection. In the case of lower frequencies, only two fundamental guided Lamb modes (the S0 and A0) exist. Due to their high sensitivity in defective regions of structures, guided Lamb waves are widely used for the inspection of different types of defects such as delaminations, cracks and impact damages, etc. [3,25,26,27]. Many approaches and transducers are available for the generation of Lamb waves. Out of those, the interdigital transducers are gaining the most recognition [28,29,30,31].

Due to its small size, light weight, flat geometry, ability to work in actuation, transmission, and sensing mode, the macro fiber composite (MFC) transducer is one of the best interdigital transducers for NDT and SHM of composite structures [32,33,34,35]. The MFC transducer consists of rectangular shaped piezo ceramic rods. These rods are sandwiched between the layers of adhesive, electrodes, and polyimide film. The electrodes attached to the film form an interdigitated pattern. The electrodes transfer the applied electrical energy to/from the rods. In our research, MFC transducer of P1-type (M-2814-P1) with dimensions of 28 × 14 mm is used. The general parametric characteristics of the MFC-2814-P1 transducer are presented in Table 1 (32).

Guided Lamb waves (the A0 and S0) can be effectively transmitted and received by using an MFC transducer [34]. The S0 mode contains dominant in-plane whereas the A0 mode contains dominant out-of-plane components of the propagating waves. The inspection using MFC transducer can be easily combined with different contact and non-contact ultrasonic inspection methods for NDT and SHM of composite structures [36,37]. The MFC transducers can be easily glued or embedded within large and complex structures without damaging the surface [38]. In aerospace applications, the embedded MFCs are frequently used for generating and harvesting ultrasonic wave energy, SHM of a structure, and detecting defects and damages due to impact [35,39]. MFCs can control the twisting motion of aircraft wings as well as the airfoils’ aerodynamic shaping [40,41]. Hence, it can increase the efficiency of an aircraft by improving its aerodynamic performance. In comparison to active fiber composite (AFC), MFC has a high fiber volume fraction which ensures its high stiffness and performance. Moreover, MFCs have better actuation performance compared to the most common piezoceramic actuators [35,40,41].

Although interdigital transducers are widely used for the transmission and reception of ultrasonic GWs, the dispersive nature and multi-modal behavior possessed by Lamb waves are the limiting factors for their adaptation and utilization in SHM. To ensure the effective application of a transducer for the inspection of a specific structure, the directivity of a transducer is one of the key parameters. Knowing the transducer directivity, the following amendments/adaptations can be performed [38]:

- The position of a transducer on the structure under inspection can be determined.
- The number/configuration of transducers can be decided.
- A specific wave mode (e.g., the S0, A0 and SH0 in LF ultrasonic) and excitation frequency can be selected for the inspection of defects.
- The best transducer for the specific application can be selected.

The analytical method is an efficient approach to calculate the directivity of transducers due to shorter processing time in comparison to the experimental or numerical analysis. An efficient 2D analytical model based on Huygens’s principle was developed in our previous research for directivity estimation of the contact-type transducer at any distance and excitation frequency with known dispersive characteristics of propagation medium and behaviour of transducer [38]. The directivity patterns of the S0, A0, and fundamental shear-horizontal mode (SH0) for the P1-type MFC transducer glued on Al alloy plate were successfully estimated by this model and the obtained results showed a good compromise with the experimental results [38]. However, the correct wave patterns under the transducer region and their effect on directivity patterns at specific frequencies were not considered in our previous work [38]. In the previous model, the amplitude variations of the excitation signal were considered a fixed value for the directivity estimations at different frequencies. A similar assumption was considered by another researcher using numerical modelling [33].

The objective of this work is to analytically analyse the wave patterns under the region of an MFC transducer glued on isotropic medium and validate by the experimental investigation, which in turn improves the 2D analytical model for the estimation of directivity patterns. The P1-type MFC transducer with dimensions (28 × 14 mm) was glued on a 2 mm thick Al alloy plate. The wave patterns under the transducer region were analyzed analytically in order to improve the previously developed analytical model [38] for the accurate analysis of the directivity patterns. We showed that wave patterns of the excitation signal are different at different frequencies under the transducer region. Hence, the frequency-dependent amplitude correction factor is estimated and included in the model. We also propose a new experimental technique to validate the wave patterns and amplitude correction factors calculated analytically. In the proposed measurement technique, MFC transducer was glued on one side of Al plate and scanning was performed on the opposite side of the plate under the region of MFC transducer. The experiment was performed by using the low-frequency (LF) ultrasonic system developed by Ultrasound Institute, Kaunas University of Technology [36,38,42]. The point-type piezoceramic transducer operating in thickness mode was used in the experimental analysis for recording the Lamb waves. The receiving transducer was more sensitive to the out-of-plane wave components.

Hence, in this research, the improvement in the directivity of only A0 mode is discussed. The calculated amplitude correction factor based on the wave patterns under the region of MFC transducer was included in the analytical modeling. The analytical solution was verified by the experimental analysis, which clearly showed a significant improvement in the directivity pattern of the A0 mode as compared to the previously obtained results.

Section 2 of this article illustrates the detailed description of a problem. Section 3 presents the calculation of the modified amplitude factor based on the wave patterns along the region of MFC transducer. The verification of the calculated amplitude factor by the experimental analysis has been presented in Section 4. A comparison of the results obtained by an analytical model and experimental investigation has been performed in Section 5 followed by the conclusive remarks in Section 6.

## 2. Description of a Problem

**Figure 1:**
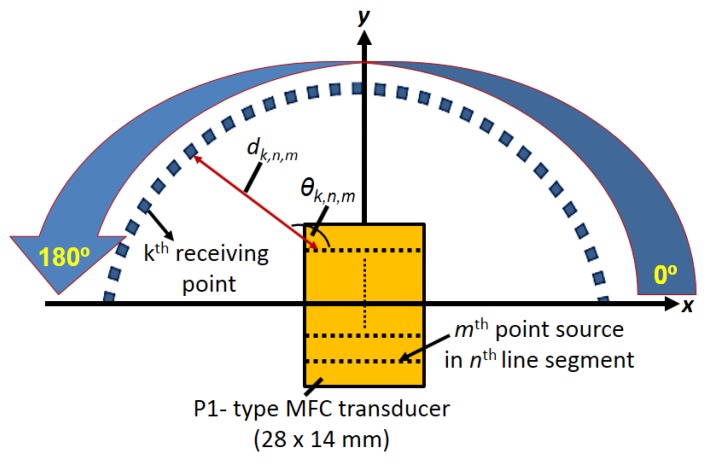
2D analytical model schematic for estimation of the directivity of P1 MFC transducer [38].

According to the previously developed 2D analytical model based on Huygens’s principle [38], the P1-type MFC transducer was considered as the number of line segments with distributed point sources along with its structure. The arbitrary points along the angles from 0° to 180° at a specified distance were considered as receiving elements. The schematic of the model is presented in Figure 1.

At each receiving point, the signals propagating from all point sources were calculated and integrated in order to calculate the received signals along the angular region. The received signal spectrum can be expressed as [38]:

(1) $$U_{R,k}\left(f,\theta_{k}\right)=\overset{N}{\underset{n=1}{\sum }}\overset{M}{\underset{m=1}{\sum }}U_{EC}\left(f\right)\cdot H_{T}\left(f,\;d_{k,n,m},v_{ph}\right)\cdot \frac{1}{\sqrt{d_{k,n,m}}}$$

where *k* is the number of receiving element (*k* = 1, 2,…*K*); *θ_k_* is the angle between the *k*^th^ receiving point and origin (*θ_k__=_* [(*k*−1)·*dθ*]; *dθ* is the angular separation between receiving elements); *n* is line segment (*n* = 1, 2,…*N*); *m* is point source (*m* = 1, 2,…*M*); *H* (*f*, *d_k,n,m_*, *v_ph_*) is the transfer function [*H* (*f*, *d_k,n,m_*, *v_ph_*)= exp (−*α*(*f*)·*d_k,n,m_*)·exp (−j2*πf d_k,n,m_*/*v_ph_* (*f*, *h*))]; *α*(*f*) is the frequency-dependent attenuation coefficient; *v_ph_* is phase dispersion velocity which depends on the thickness (*h*) of the plate and the frequency of excitation. *d_k,n,m_* is the distance from the *m*th point source to the *k*th receiving element; *U_EC_(f)* is FT of the input signal *u_EC_*(*t*); *U_R,k_* (*f, θ_k_*) is the FT of the received signal and 1/√*d_k,n,m_* is the diffraction factor corresponding to the distance.

The normalized amplitudes (*A_npp_*) along the polar coordinates to plot the directivity pattern is expressed as:

(2) $$A_{npp}\left(\theta_{k}\right)=\left[\frac{\max\left({\mathrm{FT}}^{-1}\left[U_{R,k}\left(f,\theta_{k}\right)\right]\right)-\min\left({\mathrm{FT}}^{-1}\left[U_{R,k}\left(f,\theta_{k}\right)\right]\right)}{\max\left(\max\left({\mathrm{FT}}^{-1}\left[U_{R,k}\left(f,\theta_{k}\right)\right]\right)-\min\left({\mathrm{FT}}^{-1}\left[U_{R,k}\left(f,\theta_{k}\right)\right]\right)\right)}\right]$$

**Figure 2:**
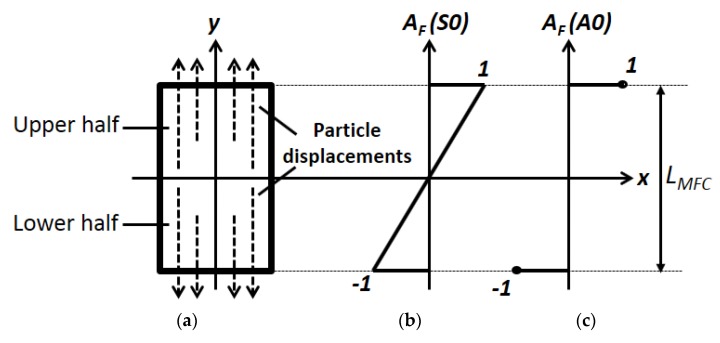
Particle displacements in P1-type MFC (28 × 14 mm) along with its length (**a**) and the amplitude correction factor (*A_F_*) by previously developed model for the S0 (**b**) and the A0 mode (**c**).

The excitation signal was multiplied by the correction factor (*A_F_*) corresponding to the particular Lamb wave mode (the S0, A0 or SH0) for the directivity estimation [38,43]. The approximated value of amplitude correction factor (*A_F_*) in the model was considered depending on the behaviour of the P1-type MFC transducer for each of the wave modes. As P1 type MFC operates in elongation mode as shown in Figure 2a, *A_F_* was considered as linearly increasing value from 0 at center up to 1(−1) at edges for upper/lower half-sections along the length (*L*_MFC_) of MFC transducer for S0 mode (Figure 2b). On the other hand, *A_F_* contained only two maximum labels with opposite polarities at the edges in the case of the A0 mode as presented in Figure 2c. It should be noted that 1 or −1 are the maximum labels and can be replaced by any numerical value.

Hence, the dependence of *A_F_* on the excitation frequency and operative wavelength was not considered in the previous model. Although the directivity patterns of the A0 mode obtained by considering *A_F_* using this approach showed significant similarity with the experimental results [38] at 80 kHz and 220 kHz excitation signals, there was still scope for improvement. The detailed specifications about the set-up and scanning procedure to estimate the directivity patterns by experimental analysis are described in [38].

**Figure 3:**
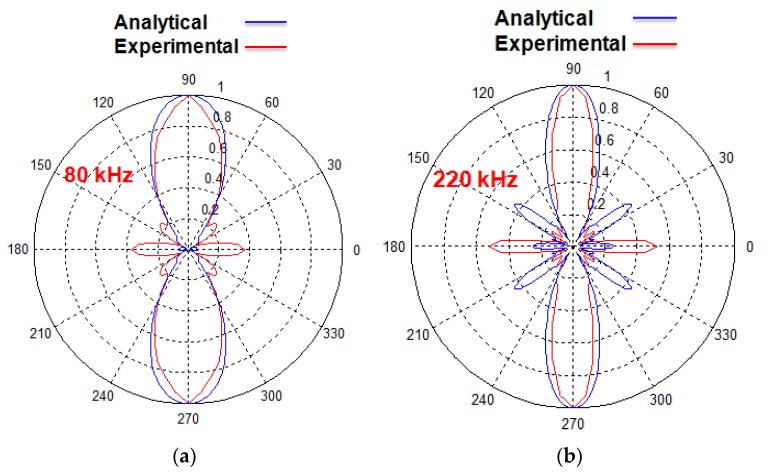
Directivity patterns of the A0 mode of P1-type MFC (28 × 14 mm) transducer at 300 mm distance from the center of a transducer without considering the appropriate wave patterns at 80 kHz (**a**) and 220 kHz (**b**).

The directivity patterns of the A0 mode calculated by a previous analytical model and the experiment at 300 mm from the center of the transducer with 80 kHz and 220 kHz excitations are shown in Figure 3 [38]. It can be clearly observed from Figure 3 that the number of side lobes in analytical results is equal to the experimental results but there is a significant difference in shapes. This is due to the fact that the signal distributions (amplitude correction factor (*A_F_*)) along the structure of the MFC transducer were not considered correctly. The numerical model developed by Haig et al. for the estimation of directivity patterns of MFC on steel plate also had a similar limitation with the amplitude correction factor [33]. Therefore, this research aims to find the accurate amplitude correction factor for the P1-type MFC transducer according to the wave patterns in its structure along the length, which in turn could improve the analytical modelling for the prediction of directivity patterns. Only the A0 mode is considered in this work due to the very high sensitivity of receiving point-type piezoceramic transducer for out-of-plane radiations.

## 3. Modified Amplitude Correction Factor (AF)

The amplitude correction factor is calculated by combining the information of wave patterns of the A0 and behavioral characteristics of P1-type MFC transducer. The wave patterns or signal distributions in the region of MFC transducer depend on the operating wavelength (*λ*) of the excitation signal along its length. The wavelength (*λ*) can be expressed by:

(3) $$\lambda =\frac{v_{ph}}{f}$$

where *v_ph_* is the phase velocity of propagation (Al alloy in our case of consideration) and *f* is the frequency of excitation signal.

The number of wavelengths (*N_λ_*) along the length (*L*) of P1-type MFC (*L* = 28 mm) can be expressed by:

(4) $$N_{\lambda }=\frac{L}{\lambda }$$

Due to the symmetry of the P1-type MFC structure and its operation in elongation (d33) mode, the approximated number of positive and negative peaks of the signals along the region of MFC transducer should be 2*N_λ_*. Therefore, rather than only two levels (Figure 2c), the profile of amplitude correction factor (*A_F_*) for the A0 mode can be represented by *D_λ_* discrete amplitude points in positive and negative directions alternatively and equispaced along the length of MFC.

A number of discrete levels must be an integer number, *D_λ_* can be expressed as:

(5) $$D_{\lambda }=\mathrm{ceil}\;\lceil 2N_{\lambda }\rceil$$

where ‘ceil’ denotes a ceiling function that maps the real number to least integer greater than or equal to the number.

The spacing between the discrete values (∆) of *A_F_* will be given by:

(6) $$\Delta =\frac{L}{D_{\lambda }-1}$$

Hence, an amplitude correction factor of excitation signal in the analytical modelling can be expressed by two different mathematical functions depending on the number of discrete levels (i.e., even or odd) along the length of MFC transducer. The two cases are illustrated as follows:

(7) $$A_{F}=\{\begin{array}{c} \delta \left(y\right)+\overset{\frac{D_{\lambda }-1}{2}}{\underset{p=1}{\sum }}\left[\delta \left(y-2p\Delta \right)-\delta \left(y-\left(2p-1\right)\Delta \right)\right],\;\mathrm{if}\;D_{\lambda }\;\mathrm{is}\;\mathrm{odd}\;\\ \overset{\left(\frac{D_{\lambda }}{2}\right)-1}{\underset{p=0}{\sum }}\left[\delta \left(y-2p\Delta \right)-\delta \left(y-\left(2p+1\right)\Delta \right)\right],\;\mathrm{if}\;D_{\lambda }\;\mathrm{is}\;\mathrm{even}\; \end{array}$$

where *y* is the longitudinal axis of MFC transducer; *p* is the discrete number depending on *D_λ_* (*p* = 1, 2… (*D_λ_*−1)/2 if *D_λ_* is odd and *p* = 1, 2… (*D_λ_*/2)−1 if *D_λ_* is even); *δ*(*y*) denotes the unit impulse signal).

**Figure 4:**
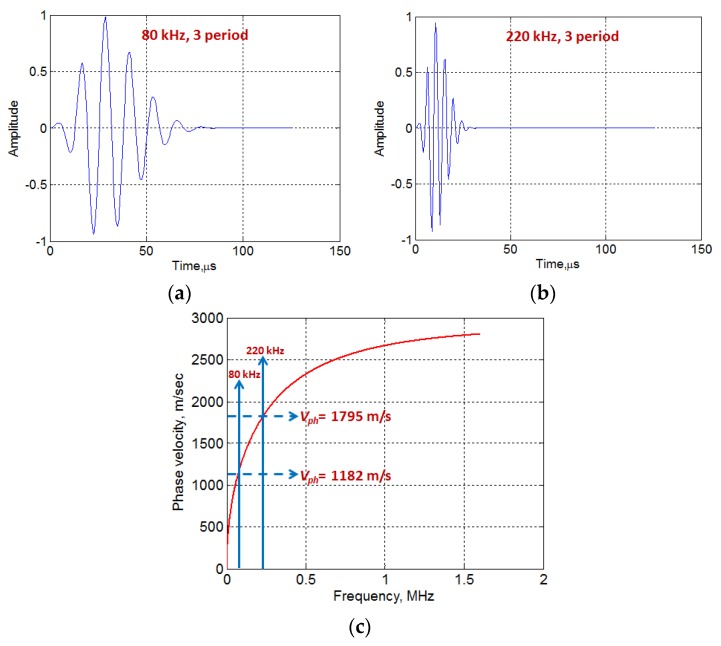
80 kHz, 3 period (**a**) and 220 kHz, 3-period excitation signals (**b**) with Gaussian symmetry and the phase velocity dispersion curve of the A0 wave mode in 2 mm Al alloy plate (**c**).

Two different excitation signals i.e., 80 kHz, 3-period and 220 kHz, 3-period with a Gaussian shape as shown in Figure 4a,b were considered in the analysis. The dispersion characteristics of the A0 mode in 2 mm Al plate were estimated using the computational package “Disperse” [44]. The phase velocity at 80 kHz and 220 kHz were observed as 1182 m/sec and 1795 m/sec, respectively, as shown in the dispersion curve (Figure 4c).

Hence, the wavelength (*λ*_80_ and *λ*_220_) and a number of wavelengths (*N_λ_*_80_ and *N_λ_*_220_) along the length and under the region of MFC transducer at 80 kHz and 220 kHz frequencies can be calculated by using Equations (3) and (4).

(8) $$\lambda_{80}=14.78\;mm;\lambda_{220}=8.16\;mm;\;N_{\lambda 80}=1.89;N_{\lambda 220}=3.43$$

**Figure 5:**
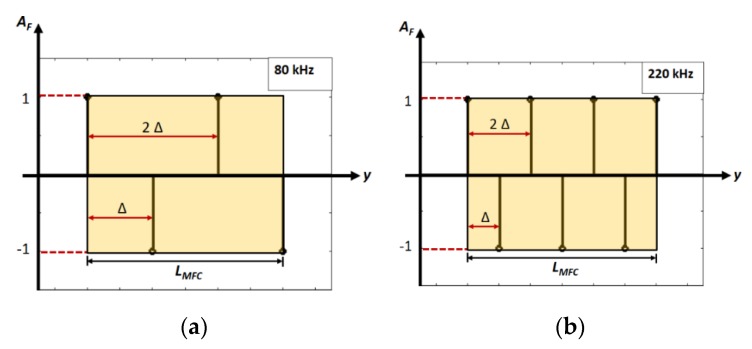
Amplitude correction factor *A_F_* at 80 kHz (**a**) and 220 kHz (**b**) along the length of MFC transducer for the A0 mode.

Thus, the approximated number of discrete positive and negative amplitudes (*D_λ_*) under the transducer region along its length can be calculated from Equation (5) as four and seven (corresponding to the excitation frequencies of 80 kHz and 220 kHz respectively. The schematic of modified amplitude correction factor *A_F_* for the A0 mode is presented in Figure 5a,b in the case of 80 and 220 kHz excitation signals respectively. The *A_F_* will have the following discrete values under the structure/region of MFC along its length:

- In the case of 80 kHz frequency, *A_F_* will have the four discrete values (i.e., two with the same polarity and two with opposite polarity). The spatial separation (∆) between the discrete values (Equation (7)) will be equal to 9.33 mm.
- Similarly, *A_F_* will contain seven discrete values (i.e., four with the same polarity and three with opposite polarity) with the excitation frequency of 220 kHz. The spatial separation (∆), in this case, will be 4.67 mm.

After including the modified amplitude correction factor, the directivity pattern can be estimated by the analytical model [38].

## 4. Experimental Validation

**Figure 6:**
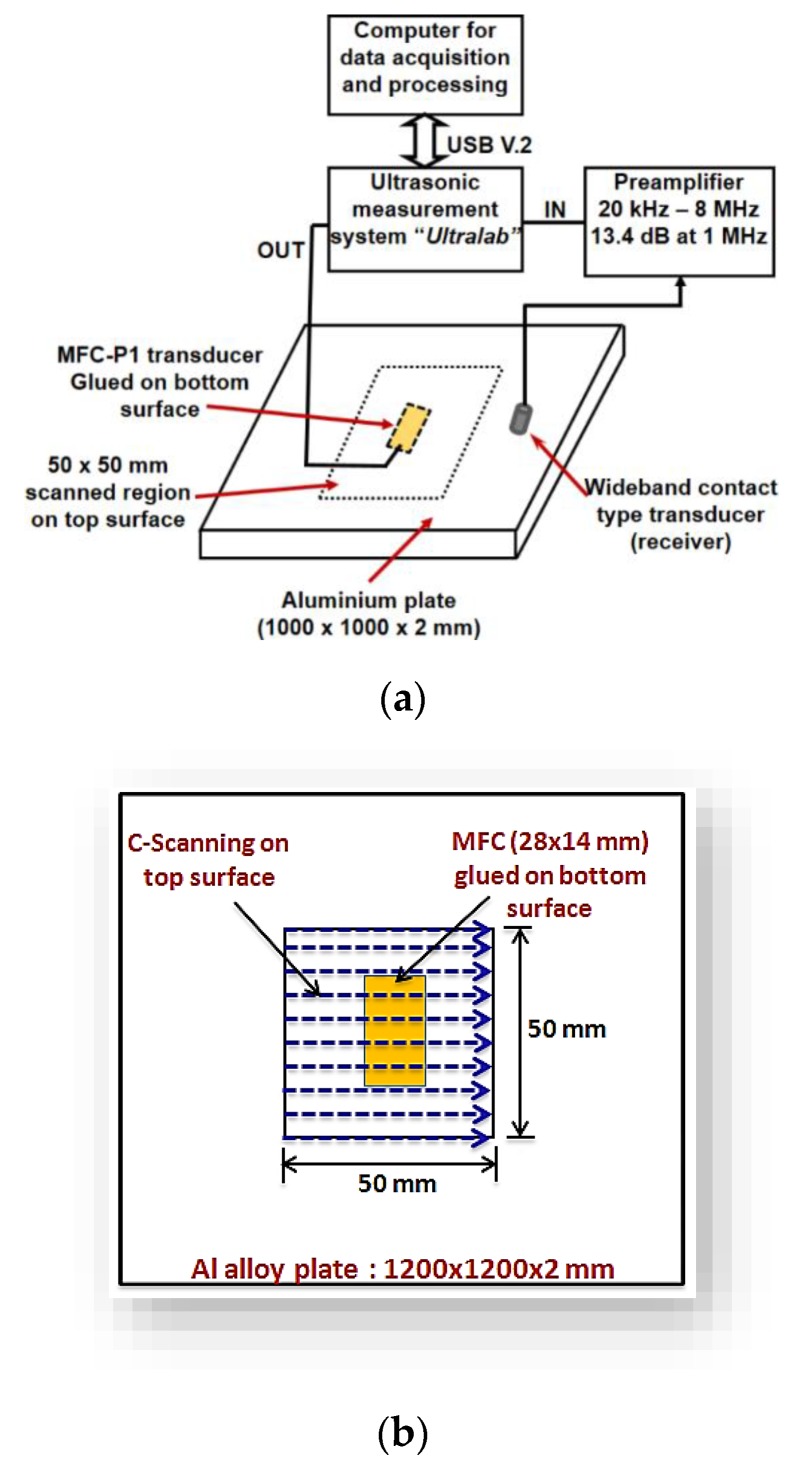
Schematic showing the experimental set-up (**a**) and C-scanning procedure in 50 × 50 mm region on the top surface with MFC glued on the bottom surface of Al alloy plate (**b**).

The new measurement technique is proposed to experimentally analyze the behaviour of MFC transducer and wave patterns along with its structure for the verification of the estimated value of *A_F_* in Section 3. The experiment was performed using the LF ultrasonic system (“Ultralab”) developed by Ultrasound Research Institute of Kaunas University of Technology. The schematic of experimental investigation is presented in Figure 6a. The characteristics of the LF ultrasonic system are described in Table 2.

The P1-type MFC-2814 (28 × 14 mm) transducer was glued at the centre of the Al alloy plate with dimension (1000 × 1000 × 2 mm) on one side of a plate. The scanning with a 1 mm step was performed on the opposite side of plate under the cross-sectional area of (50 × 50 mm) which also covered the region of MFC transducer as described in Figure 6b. The experiment was repeated two times to record the data in the case of two different excitation signals, i.e., 80 kHz, 3-period and 220 kHz, 3-period with a Gaussian shape for exciting the MFC transducer as shown in Figure 3a,b. The sampling frequency was 100 MHz. The wideband contact-type ultrasonic transducer (maximum −6 dB bandwidth was equal to 300 kHz) was used to record the ultrasonic signals. Glycerol was used for effective acoustic contact between the transducer and Al alloy plate. All components including the ultrasonic system used in the experimental investigation were developed by Ultrasound Research Institute of the Kaunas University of Technology.

**Figure 7:**
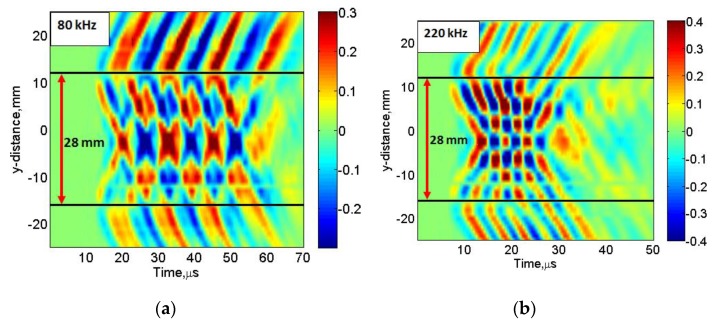
B-scan along the longitudinal axis of MFC transducer at 80 kHz (**a**) and at 220 kHz (**b**).

**Figure 8:**
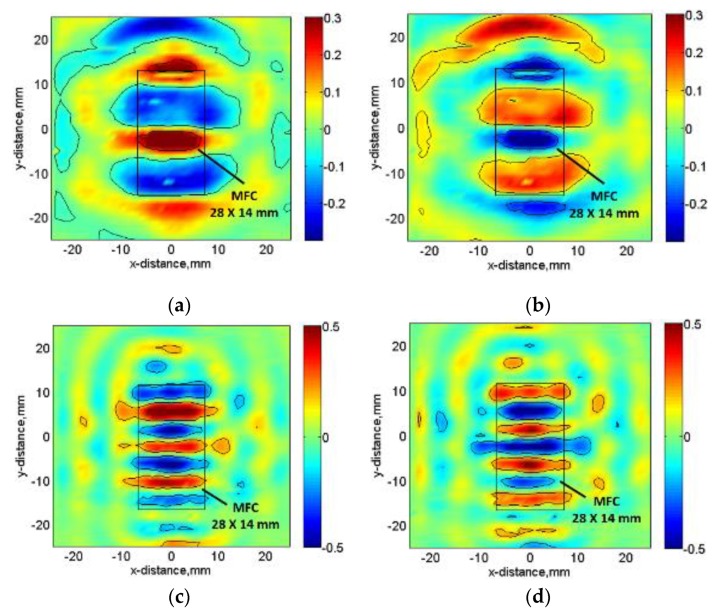
C-scan showing the wave patterns for the A0 mode: at 33 µs (**a**) and 40 µs (**b**) in the case of 80 kHz excitation; at 18.9 µs (**c**) and 21. 6 µs (**d**) in the case of 220 kHz excitation.

The B-scan images acquired along the length of MFC at 80 kHz and 220 kHz excitation frequencies are shown in Figure 7. It can be clearly observed from Figure 5a that approx. No. of discrete peaks *D_λ_* is (3.5 ≈ 4) along the length of MFC at 80 kHz frequency. On the other hand, there is approx. No. of discrete peaks equal to 7 at 220 kHz frequency as shown in Figure 5b. Therefore, these results are similar to those obtained analytically and hence validate the calculation of amplitude correction factor (*A_F_*) as described in Section 3. In order to view the two possible cases of signal peaks along the length of MFC transducer with more clear visibility, the C-scan images at 35 μs and 45 μs were acquired in the case of 80 kHz frequency. Similarly, the C-scan images at 20 μs and 24 μs were obtained for the excitation frequency of 220 kHz. The C-scan images are shown in Figure 8a–d. The time instants were chosen to show the wave patterns and number of signal peaks. The C-scan images provide a clearer visualization of the estimation of *D_λ_* and hence, the *A*_F_ in the case of 80 kHz and 220 kHz frequency respectively.

## 5. Results and Analysis

**Figure 9:**
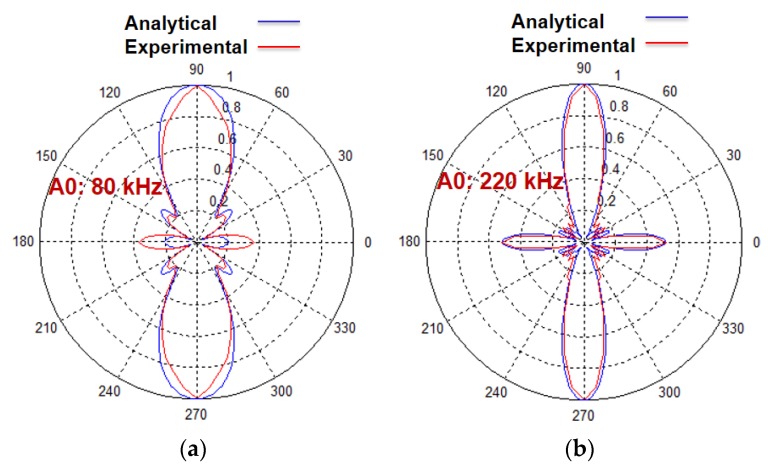
Comparison of directivity patterns of the A0 mode of P1-type MFC transducer with a modified analytical model at 80 kHz (**a**) and 220 kHz (**b**) and experimental analysis.

The estimation of amplitude correction factor analytically (Section 3) is validated by experimental analysis (Section 4). After including the modified values of amplitude correction factor *A_F_* in the analytical model, the directivity patterns of MFC transducer at 80 kHz and 220 kHz at 300 mm distance from the center of the transducer are estimated in the case of the A0 mode. The experimental investigation to obtain the directivity patterns were already performed in the previous research [38]. The directivity patterns obtained by the modified analytical model are presented in Figure 9 with their corresponding experimental results.

**Figure 10:**
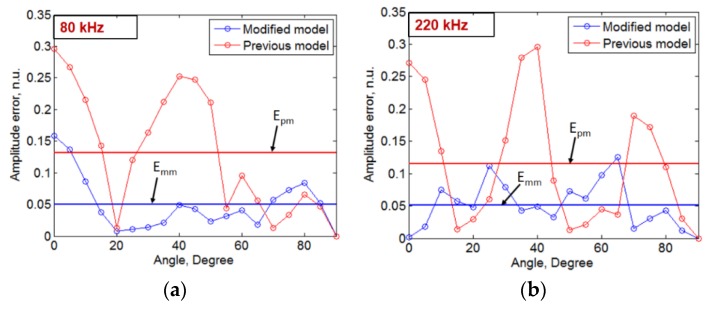
Comparison of amplitude errors in the results obtained by modified model and previously developed model in the case of 80 kHz (**a**) and 220 kHz (**b**) (*E*_pm_ -Mean error in the previous model, *E*_mm_ -Mean error in the modified model).

In comparison to the results obtained in the previously developed model [38] as presented in Figure 3, the directivity patterns obtained by the modified model show more similarities with experimental results. Therefore, the inclusion of spatial distribution of the amplitudes of excitation signal significantly improves the previously developed analytical model. This could also improve the numerical model developed by Haig et al. by resolving a similar limitation with the amplitude correction factor [33]. In order to quantitatively estimate the improvement in results as compared to the previous model, the error between the normalized amplitudes along the polar coordinates of experimental results with that obtained by previously developed model and the modified analytical model is compared. The MFC transducer is symmetric in construction. Thus, the directivity pattern along 0° to 90° with an angular separation of 5° is considered for the comparative analysis of the previous and new modified model. The absolute value of the difference between the normalized amplitudes of experimental results and the modelling results (amplitude error) along the polar axis (0° to 90°) is presented in the case of 80 and 220 kHz frequencies. The comparative results are presented in Figure 10a,b.

It is clearly observed from Figure 10a,b that the amplitude error is significantly reduced in the modified model compared to the previously developed model. At 80 kHz frequency (Figure 10a), the range of amplitude error was observed as (0–0.3) and (0–0.16) in the case of the previous model and new modified model respectively. The corresponding mean error (*E*_pm_ and *E*_mm_) in this case was estimated as 0.13 (*E*_pm_) and 0.05 (*E*_mm_), respectively. Hence, at 80 kHz frequency, the relative error in the estimation of directivity patterns by new modified model is reduced by 61.54% as compared to the previous model. In the case of 220 kHz frequency, the amplitude error lies in the range of (0–0.3) for previously developed model and (0–0.13) for the modified model. The mean error (*E*_pm_ and *E*_mm_) at 220 kHz was calculated as 0.12 and 0.05 in the case of a previously developed model and newly developed modified analytical model, respectively. The relative error at 220 kHz is reduced by 58.33% in the modified model in comparison to the previous model.

## 6. Conclusions

In this work, the accuracy of the previously developed 2D analytical model to predict and estimate the directivity pattern of the MFC transducer in the isotropic medium is increased by including the correct wave patterns of the excitation signal under the spatial region of the transducer. The wave patterns along the structure of MFC transducer are estimated analytically and validated by experimental analysis for 80 kHz and 220 kHz frequencies for the A0 mode. A new measurement technique is also proposed to analyse the spatial behaviour of the MFC transducer and wave patterns by gluing the MFC on one side of the sample and scanning on the opposite side under its region. The C-scan images under the MFC transducer and the B-scan images along the longitudinal axis of the MFC transducer were obtained at different frequencies.

In this way, we showed the dependency of amplitude correction factor on excitation frequency and included it in the present model. The P1-type MFC transducer and 2-mm thick Al alloy medium are used for a demonstration of modelling. It should be noted that dispersive phase velocity in the modelling is included by calculating the theoretical dispersion curves based on the thickness of propagating medium. In comparison to the previously developed analytical model, the error between the experimental and analytical results is reduced by 61.54% and 58.33% in the case of 80 kHz and 220 kHz, respectively. The model has significant flexibility by providing the option of selecting any isotropic propagation medium, frequency of excitation, and spatial dimensions (length and width) of transducer. In general, it is possible to include completely all distributions of the wave under the transducer. However, this leads to longer simulation time. The proposed method simplifies this task as the number of excitation points in modelling is essentially reduced compared to the case when the total spatial distribution of excitation amplitudes is taken into account.

## Figures and Tables

**Table 1:** General characteristics of MFC-P1-M2814 [32].

Features	Numerical Value
Active (length × width)	28 mm × 14 mm
Overall (length × width)	38 mm × 20 mm
Capacitance	0.61 nF
Free strain	1550 ppm
Blocking force	195 N
Operating voltage	−500 V to +1500 V
Operating bandwidth as a sensor	0 Hz to 1 MHz
Operating bandwidth as an actuator	0 Hz to 700 kHz
Maximum operational tensile strain	<4500 ppm
Linear-elastic tensile strain limit	1000 ppm

**Table 2:** Parameters of LF ultrasonic system [36,42,45].

Parameters	Numerical Value
No. of input channels	2
No. of bits of the analog-to-digital converter	10
Overall system gain (maximum)	113 dB
Ultrasonic system to computer interface	USB V.2
Frequency range	20 kHz–2 MHz
